# Interactions of *Xanthomonas* type-III effector proteins with the plant ubiquitin and ubiquitin-like pathways

**DOI:** 10.3389/fpls.2014.00736

**Published:** 2014-12-18

**Authors:** Suayib Üstün, Frederik Börnke

**Affiliations:** ^1^Plant Metabolism Group, Leibniz-Institute of Vegetable and Ornamental CropsGroßbeeren, Germany; ^2^Institute of Biochemistry and Biology, University of PotsdamPotsdam, Germany

**Keywords:** *Xanthomonas*, type-III effector, ubiquitin, proteasome, plant defense

## Abstract

In eukaryotes, regulated protein turnover is required during many cellular processes, including defense against pathogens. Ubiquitination and degradation of ubiquitinated proteins via the ubiquitin–proteasome system (UPS) is the main pathway for the turnover of intracellular proteins in eukaryotes. The extensive utilization of the UPS in host cells makes it an ideal pivot for the manipulation of cellular processes by pathogens. Like many other Gram-negative bacteria, *Xanthomonas* species secrete a suite of type-III effector proteins (T3Es) into their host cells to promote virulence. Some of these T3Es exploit the plant UPS to interfere with immunity. This review summarizes T3E examples from the genus *Xanthomonas* with a proven or suggested interaction with the host UPS or UPS-like systems and also discusses the apparent paradox that arises from the presence of T3Es that inhibit the UPS in general while others rely on its activity for their function.

## INTRODUCTION

The bacterial genus *Xanthomonas* consists of a large group of Gram-negative plant pathogenic bacteria comprising 27 species that infect a wide range of economically important crop plants, such as rice, citrus, banana, cabbage, tomato, pepper, and bean (Ryan et al., 2011). The infection strategies of various *Xanthomonas* species and pathovars are adapted to their different hosts and also exhibit tissue specificity (Ryan et al., 2011). For example, *Xanthomonas campestris* pv. *campestris* and *X. campestris* pv. *musacearum* invade through the vascular system and spread systematically whereas *X. campestris* pv. *vesicatoria* and *X. citri* pv. *citri* colonize the intercellular space (Buttner and Bonas, 2010). The broad host range of the *Xanthomonas* species and the adaptation to different tissues is also reflected in the dynamic nature of the type III effector (T3E) repertoires in a given pathovar or species. To date, ∼40 T3Es of the genus *Xanthomonas* have been identified, which are divided into groups based on their sequence identities (White et al., 2009). These T3Es function as virulence and avirulence factors either by suppressing PAMP-triggered immunity (PTI) or through the recognition by host immune receptors (Resistance proteins) and subsequent elicitation of the so called effector-triggered immunity (ETI; Jones and Dangl, 2006). Although, T3Es are assumed to contribute to virulence of *Xanthomonas*, host cellular targets and biochemical activities for many effectors remain unknown.

**FIGURE 1:**
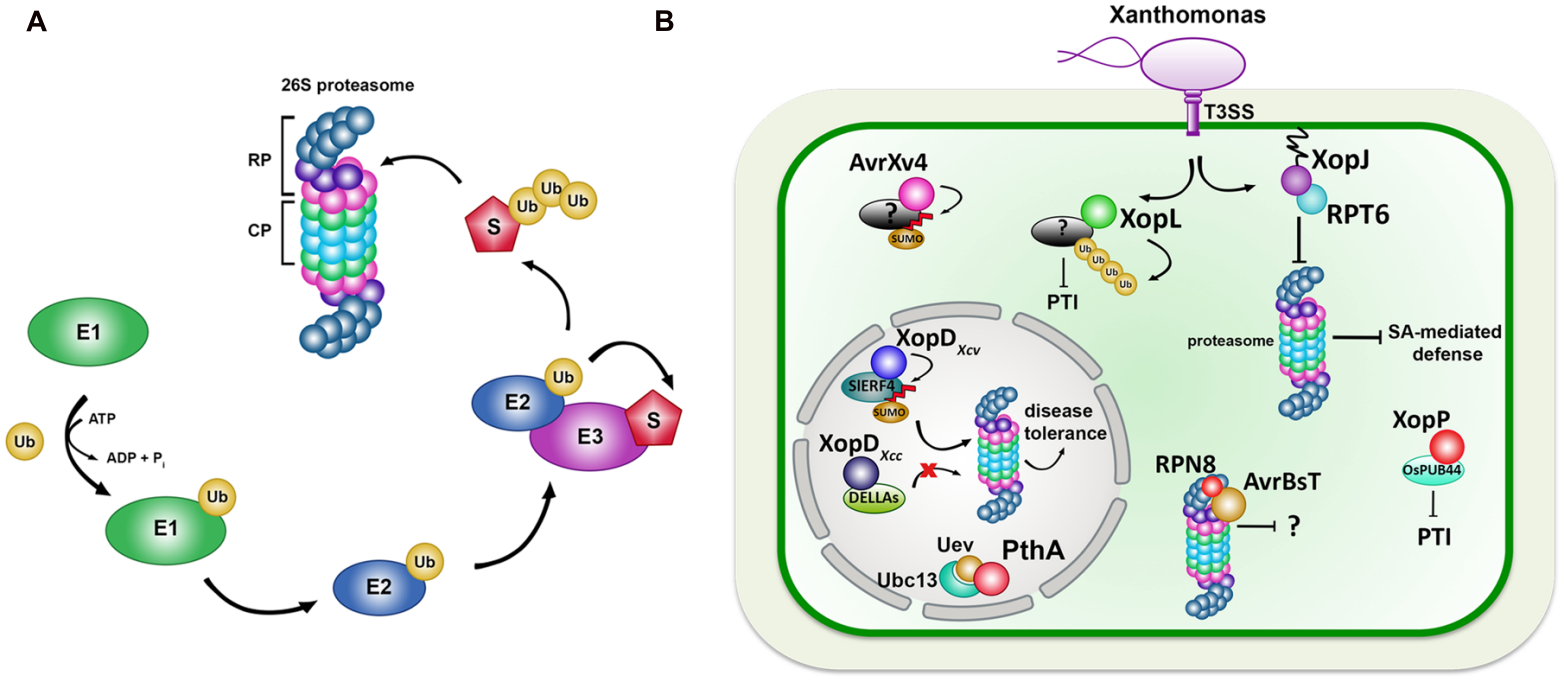
**(A)** The ubiquitin–proteasome system (UPS) and its role during plant-pathogen interactions. Ubiquitin–proteasome cascade. Activated ubiquitin binds to E1 and is transferred to the ubiquitin-conjugating enzyme (E2). The E2 carries the activated ubiquitin to the ubiquitin ligase (E3), which facilitates the transfer of the ubiquitin from the E2 to a lysine residue in the target protein (S). Poly-ubiquitinated target proteins are degraded by the 26S proteasome, consisting of a 19S regulatory Particle (RP) and 20S core subunit (CP). **(B)** *Xanthomonas* Type III effectors targeting ubiquitin and ubiquitin-like pathways. XopJ targets proteasome subunit RPT6 to inhibit the proteasome, leading to an attenuation of SA-dependent defense signaling. XopL was identified as a novel E3 Ubiquitin ligase, possibly ubiquitinating unknown target proteins leading to the suppression of PTI. AvrBsT associates with proteasome subunit RPN8 and is likely to affect proteasome function. AvrXv4 desumoylates unknown target proteins inside the plant cytoplasm. *X. citri* effectors PthA2/3 interact with the ubiquitin-conjugating enzyme complex formed by Ubc13 and ubiquitin-conjugating enzyme variant (Uev) to inhibit ubiquitination required for DNA repair. XopD from Xcc8004 targets DELLA proteins to protect them from gibberellin (GA)-induced proteasome-dependent degradation. XopD from Xcv 85-10 desumoylates tomato transcription factor SlERF4 leading to its proteasome-dependent degradation. XopP*_Xoo_* binds to OsPUB44 from rice to suppress PTI.

The ubiquitin–proteasome system (UPS) is involved in a broad array of cellular processes, such as signaling, cell cycle, vesicle trafficking, and immunity (Vierstra, 2009). Selective protein degradation by the UPS proceeds from the ligation of one or more ubiquitin proteins to the ε-amino group of a lysine residue within specific target proteins catalyzed by E1, E2, and E3 enzymes (**Figure 1A**). The ubiquitylated target protein is then recognized by the 26S proteasome for degradation. The 26S proteasome itself is a 2.5 MDa ATP-dependent protease complex composed of a 20S core protease (CP) and two 19S regulatory particles (RPs), each of which contains a lid and a base subunit (**Figure 1A**).

Beyond its role in marking target proteins for degradation via the 26S proteasome, ubiquitination can regulate cellular signaling processes. Mono-ubiquitination or multi-ubiquitination is associated with endocytosis, protein sorting, gene expression, and various other cellular pathways (Mukhopadhyay and Riezman, 2007). In addition to ubiquitination, ubiquitin-like modifications, such as SUMO (small ubiquitin-related modifier), play an essential role in various cellular functions. Similar to the ubiquitination pathway, sumoylation requires an E1-E2-E3 enzyme cascade to conjugate SUMO to the target protein. Sumoylation can affect localization, protein-protein interaction, and stability of the modified protein (Vierstra, 2012).

During the past few years, evidence has emerged that ubiquitin-and ubiquitin-like pathways play a major role in immunity and hence are subverted by bacterial pathogens in animal and plant hosts (Boyer and Lemichez, 2004; Perrett et al., 2011; Marino et al., 2012). Several components of the UPS were identified as regulators of plant immunity during PTI and ETI, such as pepper E3 ligase *CaRING1* that is induced upon *Xanthomonas* infection and is required for the activation of cell death (Lee et al., 2011). Moreover, recent studies identified that members of the U-box E3 ligase family are negative regulators of PTI (Trujillo et al., 2008; Stegmann et al., 2012). A direct connection between the UPS and ETI was shown by the fact that the accumulation of certain resistance proteins is controlled by the ubiquitin-mediated degradation via the 26S proteasome(Furlan et al., 2012).

Considering the involvement of the UPS in plant defense mechanisms, co-evolution has selected for T3Es and toxins that can manipulate ubiquitin and ubiquitin-like pathways in order to interfere with induced defense responses. The best characterized effector proteins or toxins with respect to exploitation of the UPS can be found in *Pseudomonas syringae* pv. *tomato*, a bacterium that causes bacterial speck disease on tomato plants. Some of these effectors mimic E3 ligases, e.g., AvrPtoB, to suppress both PTI and ETI events (Abramovitch et al., 2006; Janjusevic et al., 2006), whereas others, such as HopM1 promote ubiquitination of its target protein to inhibit certain induced defense responses (Nomura et al., 2006). A more direct way to subvert the UPS is achieved by SylA, a secreted toxin from *P*. *syringae* pv. *syringae,* which directly targets the catalytic subunits of the 26S proteasome to inhibit its activity and to suppress plant immune reactions (Groll et al., 2008; Schellenberg et al., 2010; Misas-Villamil et al., 2013).

In recent years, it has become evident that the UPS has a major role during the interaction of *Xanthomonas* with its plant hosts. Therefore, this mini review summarizes the current knowledge about T3Es of different *Xanthomonas* species with a demonstrated effect on ubiquitin and ubiquitin-like pathways. Possible virulence functions and conflicting actions of T3E proteins promoting or inhibiting the ubiquitin pathway are discussed.

## T3Es FROM *Xanthomonas* SPECIES INTERACTING WITH THE HOST UPS

The dual roles of UPS components in defense and development render them to be vulnerable targets for exploitation during infection. Several T3Es from *Xanthomonas* species have been shown or suggested to interact with components of ubiquitin and ubiquitin-like pathways of the host plant in a positive or negative manner (summarized in **Table 1**; illustrated in **Figure 1B**).

**Table 1:** *Xanthomonas* effectors interacting with the host UPS.

Effector	Activity/domain	Target	Role during infection	Reference
AvrXv4	deSUMOyation	?	?	Roden et al. (2004)
AvrBsT	Acetyltransferase/SUMO-protease	ACIP1, SnRKl, RPN8	Suppression of ETI/PTI	Szczesny et al. (2010)
XopJ	Cysteine protease?	RPT6	Inhibition of SA signalling	Üstün et al. (2013)
XopD_Xcv85-l0_	deSUMOyation	SLERF4	Suppression of ethylene responses	Kim et al. (2013)
XopD_Xcc8004_	deSUMOylation? deubiquitination?	DELLAs	Disease tolerance; repression of ROS	Tan et al. (2014)
XopL	E3 ubiquitin ligase	?	Suppression of PTI	Singer et al. (2013)
PthA2/3	TAL	Ubcl3/Uev	Interference with DNA repair mechanisms	Domingues et al. (2010)
XopI	F-box domain	?	?	Schulze et al. (2012)
XopP	Unknown	OsPUB44	Suppression of PTI	Ishikawa et al. (2014)

### EFFECTORS INTERACTING WITH UPS COMPONENTS

XopJ is a type III effector of *X. campestris* pv. *vesicatoria* (strain 85-10), although a highly similar sequence is also found in the genome of *X. campestris* pv. *malvacearum*. Apart from that, close homologs are also present in *Pseudomonas* spp., including *Pseudomonas avellanae*, *P. syringae* pv. *actinidiae*, *P. syringae* pv. *lachrymans* and appear to function at least in part in a XopJ-like manner (Üstün et al., 2014). XopJ belongs to the widely distributed YopJ-effector family of cysteine proteases/acetyltransferases (Hotson and Mudgett, 2004; Lewis et al., 2011). Members of this diverse T3E family are present among both plant and animal pathogenic bacteria. Based on structural similarities to cysteine proteases from adenovirus, the archetypal member of this effector family, YopJ from *Yersinia pestis,* was originally assigned to the CE clan of C55 peptidases (Orth et al., 2000). Proteases in this clan share a catalytic triad as a characteristic feature, consisting of the amino acids histidine, glutamic/aspartic acid, and a cysteine. Although recent studies demonstrated that YopJ and other members of this effector protein act as acetyltransferases on their target proteins (Mukherjee et al., 2006; Tasset et al., 2010; Lee et al., 2012; Jiang et al., 2013; Cheong et al., 2014), it has also been shown for YopJ and other members (summarized below) that these T3Es display de-sumoylating and de-ubiquitinating activities, implying that the YopJ effector family plays a role in manipulation the UPS. Initially, XopJ was identified as an T3E, as its expression is induced dependent on hrpG that controls the expression of hrp genes, being essential for the pathogenicity of Xcv (Noel et al., 2003). Further *in silico* analysis of the amino-terminal part of XopJ revealed a possible myristoylation side, being responsible for the plasma membrane localization of XopJ after translocation into the host cytoplasm (Thieme et al., 2007; Bartetzko et al., 2009). Subcellular localization of XopJ is also associated with its function to block the secretory pathway dependent on its catalytic triad and thereby interfering with cell-wall based defense responses (Bartetzko et al., 2009). Further functional analysis revealed that XopJ interacts with the 19S RP subunit RPT6 (RP ATPase 6) of the 26S proteasome. XopJ is able to recruit cytoplasmic RPT6 to the plant plasma membrane leading to the inhibition of the proteasome activity. This effect is dependent on both, its myristoylation and its catalytic triad (Üstün et al., 2013). Xcv infection of susceptible pepper plants revealed that XopJ is acting as a tolerance factor, attenuating the accumulation of salicylic acid (SA) to delay host tissue necrosis in a proteasome-dependent manner (Üstün et al., 2013). XopJ-mediated inhibition of the proteasome function also interferes with other events during plant immunity, as vesicle trafficking and callose deposition are also affected by the suppression of the proteasome. This also explains the initial observation that XopJ blocks vesicle trafficking during immunity. It is presently not clear how the inhibitory effect of XopJ on the proteasome is related to the suppression of SA-mediated defense responses. Similar to what has been proposed for SylA (Schellenberg et al., 2010), XopJ might be affecting the proteasomal turnover of NPR1, the master regulator of SA signaling, to interfere with SA-dependent immunity. Future studies regarding the protein turnover of putative target proteins of XopJ will shed light on this open question and also reveal other mechanisms implicated in XopJ-triggered immunity suppression.

Given the fact that XopJ so far has only been found in Xcv 85-10 and in *X. campestris* pv. *malvacearum*, it is possible that only certain pathovars aquired this effector during evolution to directly target the host cell proteasome as a way of adaptation to different hosts. Alternatively, other *Xanthomonas* pathovars might utilize different effector proteins involving other mechanisms to target components of the UPS. This might be the case for AvrBsT from Xcv 75-3 that was identified to interact with a UPS component. Szczesny et al. (2010) identified 19S RP subunit RPN8 as a potential interaction partner of AvrBsT in a yeast-2-hybrid assay. Similar to XopJ, AvrBsT is a member of the YopJ-superfamily of cysteine proteases/acetyltransferases, sharing 35% amino acid identity to XopJ. In addition to RPN8, AvrBsT is targeting SNF1-related kinase 1 (SnRK1), an essential regulator of nutrient and stress signaling, to possibly mediate suppression of AvrBs1-triggered hypersensitive response (Szczesny et al., 2010). Intriguingly, SnRK1 is associated with the alpha4/PAD1 subunit of the 20S proteasome to mediate proteasomal binding of a plant SCF ubiquitin ligase (Farras et al., 2001). Taken together, it is possible that AvrBsT is disrupting proteasome-mediated protein turnover similar to XopJ. However, additional experiments are required to assess the role of YopJ-like effector AvrBsT in the manipulation of the UPS machinery. Recently it was demonstrated that AvrBsT displays acetyltransferase activity toward a protein associated with microtubules and immunity (Cheong et al., 2014). Whether SnRK1 or Rpn8 are targets for AvrBsT-mediated acetylation remain to be investigated.

Another example for the exploitation of the UPS by *Xanthomonas*, is the interaction of *X. axonopodis* pv. *citri* type III effectors PthA 2 and 3 with the ubiquitin-conjugation enzyme complex formed by Ubc13 and ubiquitin-conjugation enzyme variant (Uev; Domingues et al., 2010). PthA proteins belong to the AvrBs3/PthA or TAL (transcription activator-like) family that were recently identified to act as transcriptional activators in the plant cell nucleus, where they directly bind to DNA via a central domain of tandem repeats (Boch et al., 2009). Despite the fact that effectors from the TAL family have evolved to target the plant nuclear DNA and modulate host transcription, it could be possible that proteins from this large effector family might associate with other host proteins to regulate host transcription. Both PthA 2 and 3 interact with the heterodimer complex of Ubc 13-Uev, required for ubiquitination of target proteins involved in DNA repair (Domingues et al., 2010). Taken together, this is another example of a *Xanthomonas* T3E possibly hijacking the UPS to modulate host cellular pathways.

Recently, the T3E XopP from *X. oryzae* pv. *oryzae* was shown to target OsPUB44, a rice ubiquitin E3 ligase with a unique U-box domain, to suppress peptidoglycan (PGN)- and chitin-triggered immunity and resistance to *X. oryzae* (Ishikawa et al., 2014). Although the enzymatic activity of XopP remains unknown, the authors were able to show that XopP inhibits the ubiquitin E3 ligase activity of OsPUB44, leading to its accumulation *in planta* possibly due to a loss of its auto-ubiquitination (Ishikawa et al., 2014). Whether XopP inhibits the E3 ligase activity of OsPUB44 by its biochemical activity or simply by competing for the binding site with an E2 enzyme remains to be shown.

### EFFECTORS ENCODING SUMO-PROTEASES

The initial discovery that YopJ-like effectors also share limited structural similarities with the yeast ubiquitin-like Protease 1 [ULP1, also known as small ubiquitin-like modifier (SUMO) protease], led to the assumption that these effectors may act as SUMO proteases. SUMO proteases desumoylate sumo-conjugated target proteins and as sumoylation appears to be connected to pathogen attack and other stress responses, this process might be an attractive target for bacterial invaders to modulate protein functions (Hotson and Mudgett, 2004). The first evidence that *Xanthomonas* effectors mimic SUMO proteases was provided by the functional characterization of XopD (Hotson et al., 2003). In contrast to YopJ-like effectors, XopD shares high similarities with ULPs and hence is classified as a cysteine protease belonging to the C48 family of the CE clan. XopD is localized to subnuclear foci and cleaves plant-specific SUMO precursors interfering with protein sumoylation *in planta* (Hotson et al., 2003). In the nucleus, XopD is able to bind DNA and to repress the transcription of senescence- and defense-related genes leading to the attenuation of SA-dependent senescence in tomato (Kim et al., 2008). Further analysis revealed that XopD targets tomato transcription factor SlERF4 for de-sumoylation to prevent ethylene-mediated defense responses in order to enhance bacterial propagation (Kim et al., 2013). XopD interacts with SlERF4 in the nucleus and catalyzes SUMO1 hydrolysis from lysine 53. This in turn leads to the proteasome dependent destabilization of SlERF4 (Kim et al., 2013). In summary, XopD is an example of a T3E utilizing an ubiquitin-like pathway by acting as a SUMO protease to destabilize its target protein and thereby enhancing the virulence of Xcv during infection of tomato plants.

XopD is also a paradigm for strain specific functions of homolog T3Es, as XopD from *X. campestris* pv. *campestris* (8004) uses a different strategy to modulate plant immunity: XopD*_Xcc_*_8004_ targets DELLA protein RGA (repressor of ga1-3) in the nucleus to delay its gibberellin (GA)-mediated degradation via the 26S proteasome (Tan et al., 2014). As a consequence, disease symptom development is suppressed to initiate disease tolerance and promote bacterial survival. Although the authors were not able to show that XopD*_Xcc_*_8004_ is de-ubiquitinating or de-sumoylating RGA, the study strongly suggests that XopD*_Xcc_*_8004_ somehow modifies RGA to prevent its proteasome-mediated degradation (Tan et al., 2014).

Although members of the YopJ-like effectors share restricted homology to SUMO proteases, *Xanthomonas* YopJ-like effector AvrXv4 was shown to decrease the accumulation of SUMO-modified proteins in plants (Roden et al., 2004). To date, it remains unclear whether AvrXv4 possesses SUMO isopeptidase activity and which targets are possibly de-sumoylated by AvrXv4.

### EFFECTOR PROTEINS HIJACKING THE UPS BY MIMICKING EUKARYOTIC PROTEINS

Due to the lack of structural or sequence similarities to proteins with known function, enzymatic activities for T3Es of plant pathogenic bacteria have been difficult to predict. However, the determination of the crystal structure of a number of effectors from different bacterial pathogens revealed conserved structural features with components of the host UPS (Perrett et al., 2011). For instance, crystal structure determination of *Xanthomonas* T3E XopL revealed that the protein possesses a novel fold and hence belongs to a new class of E3 ubiquitin ligases (Singer et al., 2013). Structural analysis of XopL revealed similarities to T3E E3 ligases from *Salmonella* or *Shigella*, providing first cues of an E3 ubiquitin ligase activity of XopL. Further biochemical analysis confirmed this observation, as XopL exhibits E3 ubiquitin ligase activity and interacts with specific plant E2 enzymes. The E3 ligase activity of XopL is responsible for cell death induction and also for suppression of plant immunity (Singer et al., 2013).

Alongside E3 ligases, it has been shown that proteins harboring F-box motifs are implicated in protein ubiquitination. The F-box domain is a structural motif that is ∼50 amino acids long mediating protein-protein interactions (Perrett et al., 2011). F-box proteins form a heterotetrameric ubiquitin ligase complex (SCF complex), consisting of SKP1 (S-phase-kinase-associated protein 1), Cullin and F-box proteins, mediating ubiquitination of proteins targeted for proteasomal degradation (Sadanandom et al., 2012). The first evidence that F-box proteins play a major role in plant immunity was provided by the identification of the F-box protein CORONATINE INSENSITIVE 1 (COI1), which functions as a receptor for jasmonate (Xie et al., 1998). To date, only one T3E, XopI, from *X. campestris* pv. *vesicatoria* strain 85-10 containing a F-box motif was identified, based on the presence of a PIP (pathogen-inducible promoter) box in its promoter region (Schulze et al., 2012). Type-III dependent secretion and translocation of XopI was shown during the interaction of Xcv with resistant pepper plants. However, plant target(s) of XopI remain to be identified to clarify its role in the manipulation of the UPS.

## CONCLUSION

Manipulation of ubiquitin and ubiquitin-like pathways has emerged as an effective virulence strategy for pathogenic bacteria during the past years. Several *Xanthomonas* species and pathovars appear to utilize T3E proteins from widespread families such as the YopJ-like superfamily or XopD-like family to interfere with the UPS. In addition, newly identified T3E with novel structural motifs, such as *Xanthomonas* effector XopL provide further examples. Besides T3Es acting as proteasome inhibitors, others rely on proteasome activity for their function leading to an apparent contradiction. In *X. campestris* pv. *vesicatoria* 85-10, the proteasome inhibitor XopJ and the E3 ligase XopL constitute such a effector pair. This conflicting action of T3E proteins might be resolved if T3Es interfering with the UPS would act spatially separated from each other. Posttranslational myristoylation of XopJ is responsible for its plasma membrane localization (Bartetzko et al., 2009). This feature is essential for the suppression of the proteasome activity, as XopJ interacts with RPT6 at the plasma membrane and only myristoylated XopJ is able to inhibit proteasome activity (Üstün et al., 2013). It is possible that XopL might act as an E3 ligase at a different compartment and thus, action of both T3E are separated spatially. This might be the case for XopJ and XopD, another pair with contradictory functions, as XopD acts in the host nucleus and XopJ at the plant plasma membrane. Another option would be the timing of delivery by the type III secretion system of Xcv, hence avoiding conflicting actions of both effectors.

## Conflict of Interest Statement

The authors declare that the research was conducted in the absence of any commercial or financial relationships that could be construed as a potential conflict of interest.
